# Assessment of the human bone lacuno-canalicular network at the nanoscale and impact of spatial resolution

**DOI:** 10.1038/s41598-020-61269-8

**Published:** 2020-03-12

**Authors:** Boliang Yu, Alexandra Pacureanu, Cécile Olivier, Peter Cloetens, Françoise Peyrin

**Affiliations:** 1 Univ Lyon, INSA Lyon, Université Claude Bernard Lyon 1, UJM-Saint Etienne, CNRS UMR 5220, Inserm U1206, CREATIS, 69621 Lyon, France; 2 ESRF, the European Synchrotron, 38043 Grenoble, France

**Keywords:** Bone imaging, Computed tomography

## Abstract

Recently, increasing attention has been given to the study of osteocytes, the cells that are thought to play an important role in bone remodeling and in the mechanisms of bone fragility. The interconnected osteocyte system is deeply embedded inside the mineralized bone matrix and lies within a closely fitted porosity known as the lacuno-canalicular network. However, quantitative data on human samples remain scarce, mostly measured in 2D, and there are gaps to be filled in terms of spatial resolution. In this work, we present data on femoral samples from female donors imaged with isotropic 3D spatial resolution by magnified X-ray phase nano computerized-tomography. We report quantitative results on the 3D structure of canaliculi in human femoral bone imaged with a voxel size of 30 nm. We found that the lacuno-canalicular porosity occupies on average 1.45% of the total tissue volume, the ratio of the canalicular versus lacunar porosity is about 37.7%, and the primary number of canaliculi stemming from each lacuna is 79 on average. The examination of this number at different distances from the surface of the lacunae demonstrates branching in the canaliculi network. We analyzed the impact of spatial resolution on quantification by comparing parameters extracted from the same samples imaged with 120 nm and 30 nm voxel sizes. To avoid any bias related to the analysis region, the volumes at 120 nm and 30 nm were registered and cropped to the same field of view. Our results show that the measurements at 120 and 30 nm are strongly correlated in our data set but that the highest spatial resolution provides more accurate information on the canaliculi network and its branching properties.

## Introduction

Osteocytes, the most abundant bone cells, have attracted increasing attention of scientists in the recent years. Although their role has been undervalued for a long time, various recent works have evidenced their major role in bone remodeling and repair. As endocrine cells, they could have an impact on many organs^1^. For example, osteocytes could be regulators of bone resorption by secreting Receptor activator of NF-κB ligand (RANKL)^2^ and control bone formation by producing WNT1^3^. Apart from their function in bone homeostasis, osteocytes may also contribute to fat metabolism by secreting sclerostin, leading to the increase of beige adipogenesis^4^, and influence hematopoiesis by the adjustment of the endosteal microenvironment through the release of soluble factors^5^. After being entrapped in the bone matrix, the mature osteocytes are housed within lacunae and connected to each other by cytoplasmic processes hosted within channels called canaliculi. The porous network hosting the osteocyte system is called the lacuno-canalicular network (LCN). Through the LCN, the osteocytes can transport nutrients, biochemical signals and hormonal stimuli enabling information integration and interaction with other bone cells^6^.

Since the osteocyte system is believed to act as a key regulator of skeletal homeostasis, bone diseases such as osteoporosis, osteoarthritis and osteomalacia were suggested to be associated to disorganized osteocyte networks^7^. Changes of lacunar shapes were observed in osteopenia, osteoporosis and osteoarthritis based on imaging of one sample in human knees^8^. Age related changes have been documented in several studies showing a decrease in the density of lacunae and a loss of dendrites altering the correct communication between osteocytes^9^.

Nevertheless, the characterization of the LCN remains difficult today due its location embedded within the opaque, mineralized matrix and its complexity. Conventional 2D imaging techniques such as light microscopy^10,11^, transmission electron microscopy (TEM)^12^, scanning electron microscopy (SEM)^13^, and atomic force microscopy (AFM)^14^ have been previously used to investigate the LCN. Based on 2D data, lacunae are typically described as flattened ellipsoids with an average area of about 20–70 µm^2^, an average length of about 14–25 µm and an average width of about 5–10 µm^15,16^, and the canaliculi are described as channels with an average diameter of about 100‒600 nm^17,18^. However, the 2D measurements present some uncertainty because the slicing direction may bias the results due to missing information about the third dimension.

To overcome this problem, various 3D imaging techniques are explored^19^. Confocal laser scanning microscopy (CLSM) has been used to image the LCN by recording a series of 2D optical slices^20,21^. Recently osteocytes have also been observed with advanced 3D optical techniques such as third harmonic generation imaging^21^, or multiplexed 3D-confocal imaging combining different fluorescent stains^22^. Serial focused ion beam SEM (serial FIB SEM) is another imaging technique to generate 3D reconstructions of small tissue volumes by collecting a sequence of 2D images while milling the sample layer by layer with a focused ion beam^23,24^. Besides, it is possible to image the 3D structure of the LCN with Synchrotron Radiation nano-CT (SR nCT) providing an isotropic spatial resolution and a relatively large field of view^25,26^. The feasibility of ptychographic X-ray CT with synchrotron radiation was also demonstrated^27^ and recently used to visualize the LCN in rats^28^. While providing excellent spatial resolution, acquisition time is lengthy and the specimen dimensions have to fit within a reduced field of view.

After imaging, 3D parameters are measured from the images to quantify the LCN. For lacunae, these parameters typically include the lacunar density and porosity, the lacunar volume and surface, the lacunar length, width and depth, the anisotropy and the angle between their long axis and the longitudinal axis of bone^29–32^. For canaliculi, only few parameters have been described so far. The first parameter of interest is the canalicular porosity volume, which was reported for instance in rodents^23^, and more rarely in human^33^. The diameter of canaliculi has also been reported from SR nCT images^33,34^, or ptychographic images^28^, as well as the number of canaliculi per lacuna^34,35^. While in the majority of studies the lacunae and canaliculi have been evaluated separately, a recent study used the theory of complex network to assess the LCN in ovine and murine bone^36^.

Despite the increasing interest in the LCN, data on human bone remains scarce and they are generally limited to a very small number of samples on small fields of view. In addition, the available data come from a large variety of different imaging techniques at different spatial resolutions. Since each imaging modality can generally be used to acquire images at different voxel sizes, the question arises to know which spatial resolution should be used. No study has been dedicated to the effect of spatial resolution on the 3D quantification of the LCN.

In this paper, we explore the possibilities offered by X-ray phase nano-CT associated to automatic image analysis for the 3D quantification of the LCN in human bone samples. The contribution of this work is twofold: we provide new quantitative data on the LCN from 3D images acquired with an isotropic voxel size of 30 nm, and second we study the impact of the spatial resolution on the evaluation of the 3D properties of the LCN. To this aim, we use femoral diaphysis bone samples imaged with 120 nm and 30 nm voxel sizes. We propose a method to quantify and compare the results at the two spatial resolutions. We briefly recall the image analysis techniques used to extract quantitative parameters and present new developments required to handle the volumes at 30 nm. We describe a simple image registration approach to compare the quantitative results between the images at 30 nm and the corresponding registered cropped images at 120 nm. Our results show that the measurements at 120 and 30 nm are strongly correlated in our dataset but that the highest spatial resolution provides more acurate information on the canalicular network and its branching properties.

## Methods

### Sample description

Bone specimens were harvested from the left femur of seven human cadavers (female, aging 56–95 years old, 75 ± 15 y.o.). The femurs were provided by the Department of Anatomy, Medical Faculty Rockefeller, University Lyon, France, through the French program on voluntary corpse donation to science. The protocol was approved by the French Ministry of Higher Education and Research (CODECOH “*Conservation d'éléments du corps humain*” number DC-2015–2357). All experiments were carried out in accordance with the approved protocol. The tissue donors or their legal guardians provided informed written consent to give their tissue for investigations, in accord with legal clauses stated in the French Code of Public Health. No additional information regarding donor’s disease or medication history was available following the legal clauses stated in the French Code of Public Health Ethics except for an absence of hepatitis and human immunodeficiency virus. Extracted bones were wrapped in gauze soaked with saline to keep them hydrated, then stored at −20 °C until sample preparation. Transverse cross-sections of the femoral diaphysis with a height of 4 mm were first sectioned with a diamond coated blade (Isomet 4000, Buehler, Lyon). The lateral quadrant in each cross section was cut and stored in PBS. Then small samples with a size of 0.4 × 0.4 × 4 mm^3^ were cut using a water-cooled diamond precision saw (Presi Mecatome T210, Struers Diamond Cut-off Wheel EOD15) for nano CT imaging. The samples were then dried with an ascending ethanol solution for 24 h, conserved in 70% ethanol and stored at −4 °C degrees until synchrotron imaging. We denote the samples by the number #1–7 according to the increasing order of ages.

### Synchrotron radiation nano-CT

Imaging was performed using magnified X-ray phase nano-CT at the beamline ID16A of the European Synchrotron Radiation Facility (ESRF) during several sessions of beamtime. This 3D X-ray microscopy technique enables to increase imaging sensitivity thanks to the exploitation of the phase shift of the transmitted wave instead of its attenuation. Image acquisition consisted in repeating four tomographic scans at different focus-to-sample-to-detector distances^26^. Each bone sample was mounted on a Huber pin and placed on a closed-loop nanopositioning stage inside a vacuum chamber. For each tomographic scan, 2000 angular projections were recorded while rotating the sample over a range of 180°. The energy of the X-ray beam was set either to 17.05 keV or to 33.6 keV with a monochromaticity of 1%. The projections were recorded on a 2048 × 2048 lens coupled FreLoN CCD detector. The total acquisition time for one sample scanned at 4 distances was about 4 hours. The 3D images were obtained after processing the recorded projections following two steps: phase retrieval and tomographic reconstruction. Phase retrieval consisted in processing the four projections recorded at the different propagation distances for each rotation angle to obtain a phase map. Following a previous study^37^, this was performed based on an extended Paganin’s algorithm, followed by an iterative optimization. Tomographic reconstruction was then achieved by using the Filtered Back-Projection (FBP) algorithm implemented at the ESRF within the PyHST2 software^38^. Each bone sample was scanned twice at both 120 nm and 30 nm. The final 3D reconstructed volumes (2048 × 2048 × 2048 voxels) had a field of view of 61.4 μm and 245.8 μm, respectively at 30 nm and 120 nm. The volumes at 30 nm are denoted by A1‒A7, and the site-matched volumes at 120 nm denoted by P1‒P7.

### Image processing

Image analysis was performed using custom programs. All processing steps were applied to the entire (2048)^3^ volumes.

#### Segmentation of the LCN

The segmentation of lacunae and canaliculi was performed sequentially, based on a previously described method that we briefly summarized below^31^.

For the segmentation of lacunae, we first used a median filter to eliminate some speckles, which could be canaliculi or noise, with a physical size smaller than lacunae. Then a hysteresis thresholding method was applied to segment the lacunae from reconstructed volumes and obtain binary images. This method involves two thresholds, a low threshold used to achieve a high-confidence segmentation but with less objects, and a high threshold to refine the results by checking the voxels in the ambiguous region between the two thresholds. Finally, the abnormal structures with huge size corresponding to Haversian canals were filtered out by using connected components analysis.

The automatic segmentation of canaliculi required several steps. We first used a vesselness filter for enhancement of 3D tube-like structures^39^ to improve the visibility of canaliculi. This method exploits the eigenvalues of the local Hessian matrix at each voxel. The resulting vesselness filter map was then segmented based on maximum entropy thresholding. This binary image was then used as the initialization of a specific region growing method called variational region growing^40^. This method seeks to minimize an energy functional combining gray level information from the original image and shape information from the 3D vesselness filter map to segment canaliculi. This process permits to fill gaps and reconnect some canaliculi. Finally, the smallest connected components were removed to filter out residual noise.

#### Image registration

**Figure 1:**
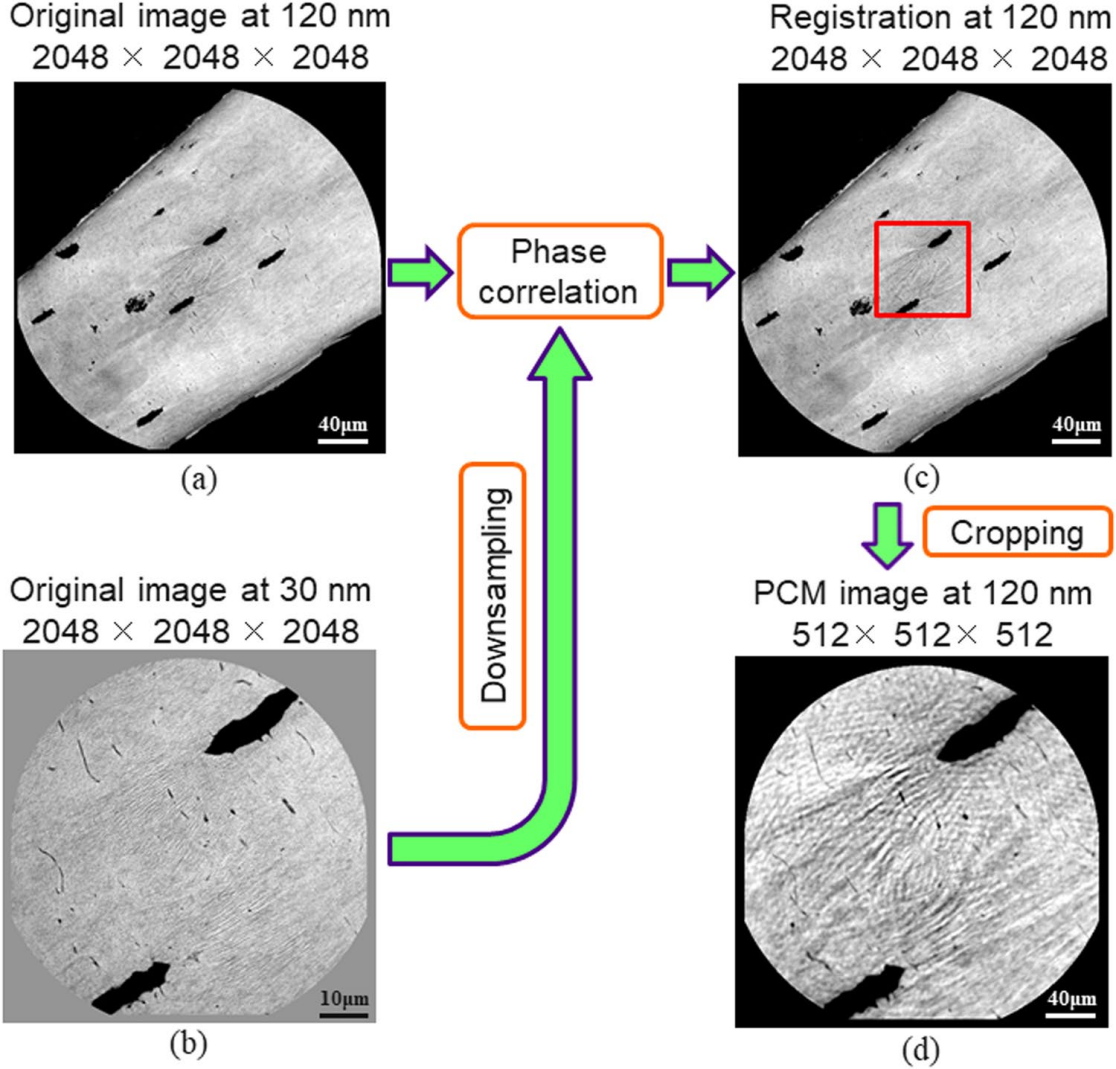
Sketch of the image registration using the phase correlation method. (**a**) The original image at 120 nm (2048^3^ voxels) and (**b**) the original image at 30 nm (2048^3^ voxels) which is first down-sampled (512^3^ voxels) are going through phase correlation. (**c**) The location of the maximum of the result provides the translation between the two images and permits to locate the common area (red square). (**d**) The 120 nm image is cropped to 512^3^ giving the same VOI than the 30 nm.

To compare the parameters extracted at 30 nm and 120 nm, we registered the corresponding reconstructed images to calculate the parameters on the same volumes of interest (VOIs). Due to our acquisition protocol, we know that the samples did not rotate but were just translated to different positions in the three dimensions, *x*, *y* and *z*. Furthermore, the change in scale is exactly known from the acquisition geometry. Therefore, the registration process is reduced to the estimation of a translation that we addressed by a phase correlation method (PCM), illustrated in Fig. 1.

For a given sample, let us denote ${I}_{1}({\bf{x}})$ and ${I}_{2}({\bf{x}})$ the reconstructed volumes at 120 nm and 30 nm respectively, where ${\bf{x}}=(x,y,z)$ are the 3D coordinates. First, we downsampled the original 2048^3^ volume at 30 nm twice in each direction yielding a 512^3^ volume with a voxel size of 120 nm, denoted by ${I}_{{2}_{D}}({\bf{x}})$. Then, this volume was zero-padded to 2048^3^, denoted by ${I}_{{2}_{DP}}({\bf{x}})$ and we calculated the phase correlation $p({\bf{x}})$ between the two volumes as^41^:

1 $$p({\bf{x}})={ {\mathcal F} }^{-1}\,\left[\frac{{\tilde{I}}_{{2}_{DP}}^{\ast }({\bf{f}}){\tilde{I}}_{1}({\bf{f}})}{|{\tilde{I}}_{{2}_{DP}}^{\ast }({\bf{f}}){\tilde{I}}_{1}({\bf{f}})|}\right]({\bf{x}}),$$

where ${\tilde{{\rm{I}}}}_{1}({\rm{f}})$ is the 3D Fourier transform of ${{\rm{I}}}_{1}({\rm{x}})$, ${\tilde{{\rm{I}}}}_{{2}_{{\rm{DP}}}}^{\ast }({\rm{f}})$ the complex conjugate of the Fourier transform of ${{\rm{I}}}_{{2}_{{\rm{DP}}}}({\rm{x}})$, where ${\bf{f}}=({{\rm{f}}}_{1},{{\rm{f}}}_{2},{{\rm{f}}}_{3})$ are the spatial frequencies and ${ {\mathcal F} }^{-1}$ the inverse 3D Fourier transform.

The peak intensity of $p({\bf{x}})$ corresponds to the translation offsets along the three axes (Fig. 1(c)). Using these offsets, we cut a 512^3^ volume from the original image ${I}_{1}({\bf{x}})$ at 120 nm. This cropped volume, denoted by ${I}_{{1}_{PCM}}({\bf{x}})$, corresponds to the same region as ${I}_{2}({\bf{x}})$ in the sample and has the same field of view of 61.44 μm (Fig. 1(d)).

This process was applied to the seven volumes at the voxel size of 120 nm. The resulting cropped volumes are called here “PCM volumes” and denoted by “P”.

### Quantitative analysis

After the segmentation of the LCN, we calculated the same lacunae and canaliculi parameters both for the original volumes at 30 nm and the site-matched volumes at 120 nm.

#### Quantification of lacunae

We first computed the number of lacunae (*Lc*.*N*), the total volume of lacunae (*Lc*.*TV*), the bone volume (*BV*), the density of lacunae (*Lc*.*N*/*BV*) and the lacunar porosity (*Lc*.*TV*/*BV*). Then, the segmented image of lacunae was labeled (i.e. assigning one label to each lacunae) and a 3D Voronoi tessellation was performed. This process divides the images into different geometrical patches, where each patch contains only one lacuna^42^. Each patch or Voronoi cell can be interpreted as the local environment of the lacuna. From this information, we measured the average volume of lacuna (Lc.V), the average volume of the Voronoi cells (Cell.V) and the local lacunar porosity (Lc.V/Cell.V).

Moreover, we calculated morphological descriptors of each lacuna by fitting it to an ellipsoid through the second-order moments matrix^43^. It provided us the length, width and depth of lacuna (*Lc*.*L*_1_, *Lc*.*L*_2_, and *Lc*.*L*_3_), as well as the anisotropy of lacuna described by the ratios between different axes (*Lc.L*_1_/*Lc.L*_2_ and *Lc.L*_2_/*Lc.L*_3_). Besides, we calculated the average surface area of the lacunae (*Lc*.*S*) and the structure model index of the lacunae (*Lc*.*SMI*) as described by Ohser^44^.

#### Quantification of canaliculi

We calculated the total volume of canaliculi (*Ca.TV*), the porosity of canaliculi (*Ca.TV*/*BV*), the average volume of canaliculi per cell (*Ca.V*), the local porosity of canaliculi measured on the Voronoi cells (*Ca.V*/*Cell.V*) and the ratio between the average volume of canaliculi and lacunae per cell (*Ca.V*/*Lc.V*). By adding the lacunae volume, we computed the total volume of the LCN (*LCN*.*TV*) and the porosity of the LCN (*LCN.TV*/*BV*) for the evaluation of the whole network. Moreover, to quantify the ramification of canaliculi, we calculated the number of canaliculi per lacuna *Ca.N*(*r*) at different distances *r* from the surface of the lacuna^45^. This calculation was based on the number of holes on the bounding surface. In a previous work of our group^45^, this surface was generated by the numerical dilation of the surface of the segmented lacuna. However, the surface of lacuna at 30 nm contains too many voxels so the 3D dilation becomes very time-consuming. Here, we propose another way to obtain the bounding surface based on the equation of the ellipsoid. An ellipsoid of center ${\bf{c}}=(xc,yc,zc)$ with axis defined by a rotation **R** can be expressed by the matrix equation:

2 $${({\bf{x}}-{\bf{c}})}^{T}{{\bf{R}}}^{T}{\bf{A}}{\bf{R}}({\bf{x}}-{\bf{c}})=1.$$

where ${\bf{x}}=(x,y,z)$ denotes the coordinates of the ellipsoid points and **A** is a diagonal matrix coding the length, width and depth of the ellipsoid. Knowing the initial length, width and depth of the ellipsoid, *L*_1_, *L*_2_, *L*_3_, we can express the isotropic dilation by a factor *r* of the ellipsoid representing a lacuna, by modifying the matrix A as:

3 $${\bf{A}}=(\begin{array}{ccc}1/{({L}_{1}/2+r)}^{2} & 0 & 0\\ 0 & 1/{({L}_{2}/2+r)}^{2} & 0\\ 0 & 0 & 1/{({L}_{3}/2+r)}^{2}\end{array}),$$

In our case, the length, width and depth of the ellipsoid and the column vectors of the rotation matrix **R** were estimated from the eigenvalues and eigenvectors of the second-order moment matrix^31^. Therefore, the dilated lacunae surface can be expressed analytically by Eq. (3) thus reducing computing time. This virtual dilatation was performed for seven increasing values of *r* from 1.2 µm to 12 µm to obtain the evolution of the number of canaliculi at different distances from the lacunae surface.

In addition, we computed the ratio between the number of canaliculi per lacuna and the surface area of lacuna located in the same cell (*Ca.N*/*Lc.S*) in order to quantify the density of canaliculi.

### Statistical analysis

We used the software Statview^®^ (SAS Institute Inc., Cary, NC, USA) for the statistical analysis. We tested if each parameter was different when computed from the 30 nm image or the corresponding PCM one. First the Kolmogorov-Smirnov (K-S) test was used to assess the normality and the *F-test* to determine the homogeneity of variances. When these conditions were verified, the paired *t*-test was used to test the difference between the two groups. If the conditions were not verified, the results were statistically tested by the Wilcoxon signed rank test. Besides, we used the Spearman correlation coefficient (*R*^2^) and the *p*-value by the Fisher’s *r* to *z* transformation to evaluate correlations between the measured parameters. Results with *p*-values under 0.05 were considered as significant.

## Results

### Image Processing

Figure 1 illustrates the results of image registration. Figure 1(a,b) show slices from the original input images (2048 × 2048 × 2048 voxels) at 120 nm and 30 nm voxel sizes, respectively. Figure 1(b) indicates the position of the cropped region within a slice at 120 nm. Figure 1(d) shows a slice of the cropped volume at 120 nm (512 × 512 × 512 voxels) restricted to the region of interest. The comparison of Fig. 1(b,d) shows that the phase correlation method finds the accurate position of the VOI in the image at 120 nm, corresponding to that in the image at 30 nm.

**Figure 2:**
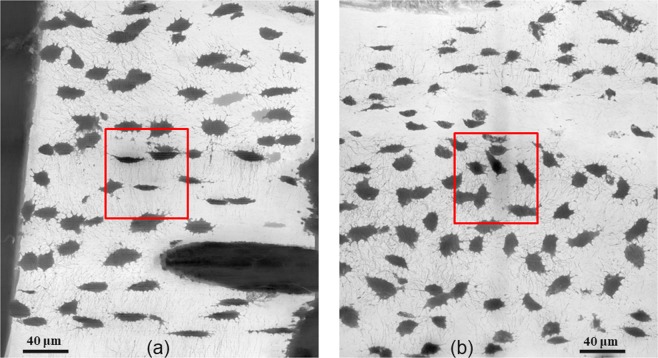
Illustration of the Minimum intensity projections along the Y-axis for samples #1 (left) and #5 (right) at 120 nm showing the full Field of View. The projection depth is 512 voxels i.e. 61.4 µm. The red square illustrates the VOI of the image at 30 nm.

**Figure 3:**
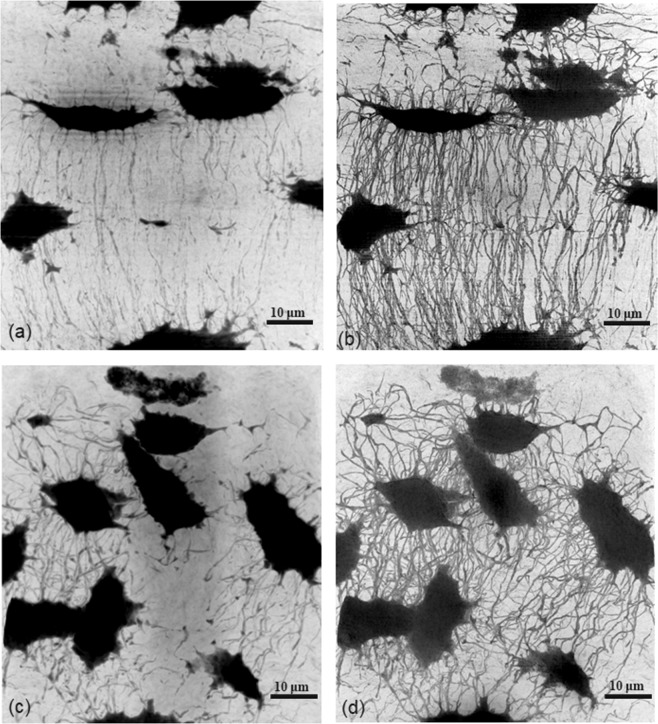
Minimum intensity projections along the Y-axis of the same VOI at 120 nm (left) and 30 nm (right). Top (**a**,**b**): sample #1 Bottom (**c**,**d**): sample #5.

**Figure 4:**
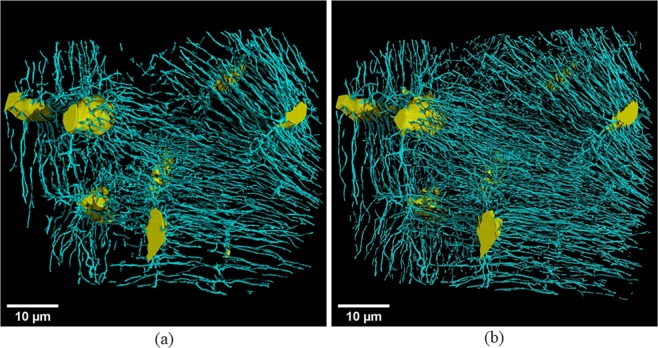
3D renderings of the segmented lacunae and canaliculi corresponding to sample #1 at 120 nm (left) and 30 nm (right).

Figure 2(a,b) show the Minimum Intensity Projections (MIPs) calculated on 512 slices along the Y-axis for two volumes at 120 nm (samples #1 and #5). The area corresponding to the position of the volume at 30 nm is overlaid in red. Figure 3 displays the MIPs of the registered regions at 120 nm (left) and 30 nm (right) for sample #1 ((a) and (b)) and sample #5 ((c) and (d)). Comparing Fig. 3(a–d), it can be observed that for the same VOI, we obtain a better detection and segmentation of canaliculi at 30 nm voxel size compared with 120 nm. The 3D visualizations of the segmented lacunae and canaliculi rendered by VGStudioMax^®^ are displayed in Fig. 4(a) (at 120 nm) and (b), (at 30 nm) respectively. We also provide movies in supplementary materials. Two movies show the 400 middle slices in volume A1 and the corresponding 100 middle slices in volume of P1 sharing the same field of view (see Supplementary Videos S1 and S2). Two other movies present 3D renderings of the entire segmented volumes A1 and P1 (see Supplementary Videos S3 and S4).

### Quantitative results at 30 nm

Table 1 reports the average and standard deviation of the morphometric parameters of lacunae calculated the seven samples at 30 nm. The average lacunar porosity was 1.10%. The average volume of each Voronoi cell was 2.6 × 10^4^ µm^3^ and the ratio between the average volume of each lacuna and its enclosing cell was 1.61%. Table 2 shows the parameters measured on canaliculi in each sample (n = 7) at 30 nm. We can see that the average canaliculi porosity was 0.35%, and that the total LCN porosity was 1.45%. The average volume of canaliculi per cell is 115.3 µm^3^ with a local canalicular porosity of 0.48%. The ratio between the volume of canaliculi and corresponding lacunae in the same Voronoi cell is 37.7% on average. Table 3 displays the quantitative results of the ramification of canaliculi. These parameters permit to assess the number of canaliculi issued from each lacuna and follow its evolution at different distances from the lacunar surface. The results show that average number of canaliculi per lacuna increases from 79.7 to 114.5. The corresponding density of canaliculi in the same cell also increases from 0.21 µm^−2^ to 0.32 µm^−2^.

**Table 1:** Morphometric parameters of lacunae at different voxel sizes.

	Lc.N	Lc.TV* (10^−5^ mm^3^)	BV (10^−3^ mm^3^)	Lc.TV/BV* (%)	Lc.N/BV (10^4^ mm^−3^)	Lc.V* (µm^3^)	Cell.V (10^4^ µm^3^)	Lc.V/Cell.V* (%)
30 nm	5.3 ± 2.1	0.18 ± 0.08	0.17 ± 0.02	1.10 ± 0.48	3.2 ± 1.2	347.8 ± 61.4	2.62 ± 0.65	1.61 ± 0.54
120 nm (pcm)	5.3 ± 2.1	0.17 ± 0.07	0.17 ± 0.02	1.01 ± 0.44	3.2 ± 1.2	315.6 ± 51.7	2.6 ± 0.6	1.47 ± 0.51
Diff	—	8.9%	—	8.9%	—	8.9%	0.9%	9.0%
		Lc.S* (µm^**2**^**)**	Lc.L1* (µm)	Lc.L2* (µm)	Lc.L3* (µm)	**Lc.L1 /Lc.L2**	**Lc.L2 /Lc.L3**	**Lc.SMI**
30 nm		369.7 ± 57.3	17.2 ± 1.8	9.4 ± 1.1	4.8 ± 0.7	1.9 ± 0.3	2.1 ± 0.5	3.1 ± 0.3
120 nm (pcm)		326.0 ± 50.0	16.7 ± 1.7	9.0 ± 1.0	4.6 ± 0.7	2.0 ± 0.3	2.1 ± 0.5	3.1 ± 0.3
Diff		11.7%	3.2%	3.7%	3.6%	0.9%	2.5%	1.9%

Lc.N ‒ number of lacunae Lc.TV ‒ total volume of lacunae (mm^3^).

BV ‒ bone volume (mm^3^) Lc.TV/BV ‒ lacunar porosity (%).

Lc.N/BV ‒ density of lacunae (mm-3) Lc.V ‒ average volume of lacuna (µm^3^).

Cell.V ‒ average volume of each Voronoi cell (µm^3^) Lc.S ‒ average surface area of lacuna (µm^2^).

Lc.V/Cell.V ‒ local lacunar porosity (%) Lc.SMI ‒ average structural model index of lacuna.

Lc.L1, Lc.L2 and Lc.L3 ‒ average length, width and depth of lacuna (µm).

Lc.L1/Lc.L2 and Lc.L2/Lc.L3 ‒ average anisotropy of lacuna.

*p-value < 0.05.

**Table 2:** Morphometric parameters of canaliculi at different voxel sizes.

	Ca.TV* (10^−5^ mm^3^)	LCN.TV* (10^−5^ mm^3^)	Ca.TV/BV* (%)	LCN.TV/BV (%)	Ca.V* (µm^3^)	Ca.V/Cell.V* (%)	Ca.V/Lc.V (%)
30 nm	0.06 ± 0.03	0.24 ± 0.10	0.35 ± 0.16	1.45 ± 0.57	115.3 ± 45.8	0.48 ± 0.20	37.7 ± 16.3
120 nm (pcm)	0.05 ± 0.03	0.22 ± 0.09	0.30 ± 0.16	1.31 ± 0.55	97.2 ± 48.3	0.41 ± 0.19	34.5 ± 16.1
Diff	17.6%	10.9%	17.6%	10.9%	17.6%	15.0%	10.7%

Ca.TV ‒ total volume of canaliculi (mm^3^) LCN.TV ‒ total volume of the LCN (mm^3^).

Ca.TV/BV ‒ porosity of canaliculi (%) LCN.TV/BV ‒ porosity of the LCN (%).

Ca.V ‒ average volume of canaliculi per cell (µm^3^) Ca.V/Cell.V ‒ local porosity of canaliculi.

Ca.V/Lc.V ‒ ratio between the average volume of canaliculi and lacuna per cell (%).

*p-value < 0.05.

**Table 3:** Number of canaliculi per lacuna at 7 different distances from the surface of the lacuna and density of canaliculi at different voxel sizes.

	Ca.N* (r = 1.2 µm)	Ca.N* (r = 3.0 µm)	Ca.N* (r = 4.8 µm)	Ca.N* (r = 6.6 µm)	Ca.N* (r = 8.4 µm)	Ca.N* (r = 10.2 µm)	Ca.N* (r = 12.0 µm)
30 nm	79.7 ± 19.2	82.7 ± 25.6	86.2 ± 29.8	85.9 ± 37.8	90.2 ± 42.6	100.0 ± 47.1	114.2 ± 57.3
120 nm (pcm)	49.7 ± 15.1	47.9 ± 19.9	49.8 ± 24.5	48.7 ± 25.6	47.2 ± 26.5	47.7 ± 27.9	49.1 ± 34.1
Diff	38.8%	44.4%	46.9%	48.8%	52.2%	56.5%	60.9%
	Ca.N/Lc.S* (µm^**−2**^**, r = 1.2 µm)**	Ca.N/Lc.S* (µm^**−2**^**, r = 3.0 µm)**	Ca.N/Lc.S* (µm^**−2**^**, r = 4.8 µm)**	Ca.N/Lc.S* (µm^**−2**^**, r = 6.6 µm)**	Ca.N/Lc.S* (µm^**−2**^**, r = 8.4 µm)**	Ca.N/Lc.S* (µm^**−2**^**, r = 10.2 µm)**	Ca.N/Lc.S* (µm^**−2**^**, r = 12.0 µm)**
30 nm	0.21 ± 0.05	0.22 ± 0.07	0.23 ± 0.08	0.23 ± 0.10	0.24 ± 0.11	0.27 ± 0.12	0.32 ± 0.14
120 nm (pcm)	0.16 ± 0.05	0.15 ± 0.07	0.16 ± 0.08	0.16 ± 0.08	0.15 ± 0.08	0.15 ± 0.08	0.16 ± 0.10
Diff	27.9%	34.7%	38.2%	39.4%	43.2%	49.4%	53.8%

Ca.N ‒ number of canaliculi per lacuna Ca.N/Lc.S ‒ density of canaliculi.

*p-value < 0.05.

### Quantification of cropped volumes at 120 nm

The same analysis was performed on the seven registered and cropped volumes at 120 nm voxel size. Table 1 also presents the lacunae parameters calculated from the cropped volumes. The average number of lacunae and density of lacunae are unchanged compared to 30 nm. The average lacunar porosity was 1.01%. Table 2 also shows the morphometric canaliculi parameters for the cropped volumes at 120 nm. The canaliculi porosity was 0.30% on average and the total LCN porosity was 1.31%. The average volume of canaliculi per Voronoi cell was 97.2 µm^3^ with a local canaliculi porosity of 0.41%. In addition, the average ratio between the canaliculi and lacuna volume within the same Voronoi cell was 34.5%. Table 3 displays the quantitative results of the ramification of canaliculi at the same seven distances from the lacuna surface as at 30 nm.

### Comparison of parameters on matched VOIs at 30 nm and 120 nm

The average relative difference between parameters measured at 30 nm and 120 nm are reported in Tables 1–3.

**Figure 5:**
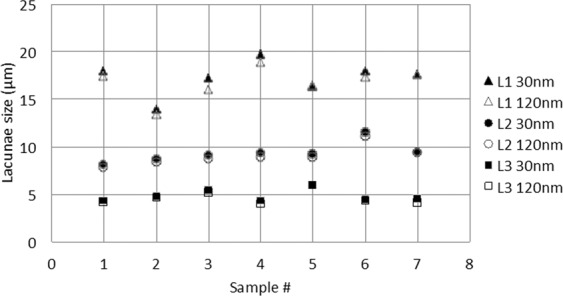
Plot of lacunae sizes Lc.L_1_, Lc.L_2_, Lc.L_3_ measured at 30 nm (black) and 120 nm (white) for the different samples.

**Figure 6:**
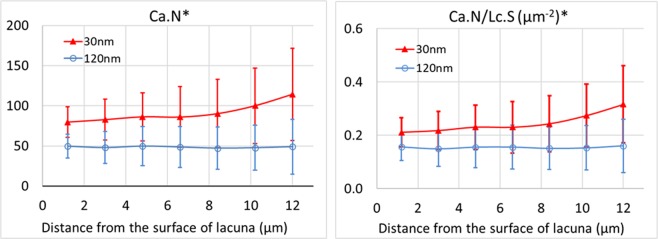
Plot of the evolution at different distances of the number of canaliculi *Ca.N* (left) and the density of canaliculi per lacunae surface *Ca.N/Lc.S* (right) at 30 nm (triangle) and 120 nm (circle) (*p-value < 0.05).

Figure 5 illustrates the values of lacunae sizes, *Lc.L*_1_, *Lc.L*_2_, *Lc.L*_3_ imaged at 30 nm and 120 nm, showing a slight increase of parameter values at 30 nm compared to 120 nm. Figure 6 shows the average number of canaliculi per lacuna and the average density of canaliculi as a function of the distance to the surface of lacunae.

Table 4 shows the Spearman correlation coefficients and the corresponding *p*-values between the parameters from the same VOIs at 30 nm and 120 nm. We wanted to check whether there are good correlations (*R*^2^ > 0.5 and *p* < 0.05) between these parameters quantified at different voxel sizes. For the parameters quantified from the volumes at 30 nm and the PCM ones, all the parameters passed the test of the homogeneity of variances. Therefore, we used the paired *t*-test for the evaluation of parameters. Between the two groups, there were no significant differences for the *Lc.N*/*BV*, *Cell.V*, *Lc.L*_1_/*Lc.L*_2_, *Lc.L*_2_/*Lc.L*_3_, *Lc.SMI*, *Ca.V*/*Lc.V*. However, the results of *Lc.TV*/*BV*, *Lc.V*, *Lc.V*/*Cell.V*, *Lc.S*, *Lc.L*_1_, *Lc.L*_2_, *Lc.L*_3_, *LCN.TV/BV*, *Ca.TV/BV*, *Ca.V*, *Ca.V/Cell.V*, *Ca.N* and *Ca.N/Lc.S* at 30 nm were significantly different from those of the PCM volumes (*p*-value < 0.05).

**Table 4:** Spearman correlation detection between parameters from the volumes at the voxel size of 30 nm and the cropped ones at 120 nm.

Parameter	*Slope*	*R*^2^	*p*-value
Lc.TV/BV	0.9198	0.9951	<0.0001
Lc.V	0.8300	0.9710	<0.0001
Lc.S	0.8207	0.8849	0.0016
Lc.V/Cell.V	0.9313	0.9755	<0.0001
Lc.L1	0.9483	0.9470	0.0002
Lc.L2	0.9249	0.9885	<0.0001
Lc.L3	1.0384	0.9713	<0.0001
Ca.TV/BV	0.9459	0.9594	0.0001
LCN.TV/BV	0.9476	0.9964	<0.0001
Ca.V	0.9979	0.8955	0.0012
Ca.V/Cell.V	0.9514	0.9542	0.0002
Ca.V/Lc.V	0.9398	0.9038	0.0010

Besides, we give the quantitative results for all the reconstructed volumes at 30 nm and the cropped volumes at 120 nm in Supplementary Tables S1 and S2, respectively. To check the ramification of canaliculi, we also show the plots of *Ca.N* and *Ca.N*/*Lc.S* for all the volumes at different voxel sizes in Supplementary Figs. S1 and S2.

## Discussion

This work reports quantitative parameters of the LCN from X-ray phase nano-CT images at two voxel sizes and assesses the effect of the spatial resolution by comparing results obtained from registered images. A voxel size of 30 nm is the highest spatial resolution that has ever been reported for the analysis of human bone samples by using this technique, even if similar spatial resolution can be reached for instance from ptychography^28^ or FIB/SEM techniques^23^. Nevertheless, the effect of the improved spatial resolution in terms of the quantitative assessment of the LCN needed to be clarified. Our data at two spatial resolutions permitted to address this question by comparing results on site-matched volumes of the same samples. Due to the acquisition protocol ensuring a scaled, rigid transformation without rotation between the two images, the phase correlation method was found suitable to register corresponding 3D images.

In total, 7 human femoral diaphysis samples were analyzed with an average of 5.3 lacunae per sample. This analysis was performed using a custom-made automatic digital image analysis program enabling to binarize the LCN and extract a number of 3D parameters. Most of the previous findings on the LCN were based on image analysis tools requiring human interaction. While this can be performed easily on few 2D images with small fields of view, scaling up to 3D images of 2048 × 2048 × 2048 voxels as in our study becomes incompatible with manual segmentation and quantification. It would require lengthy and tedious human work to delineate lacunae and canaliculi, likely with limited accuracy, and the extraction of 3D measurements from a stack of slices is not straightforward. All our parameters were measured directly in 3D space and we proposed new parameters such as those characterizing the local porosity around each lacuna using Voronoi tessellation.

The density of lacunae was found to be 32 000 mm^−3^ and was not affected by the voxel size. The results are in the range of previous studies for the density (10,000–35,000 mm^−3^)^30,31,46–48^. Concerning the morphological properties of lacunae, the average volume (*Lc.V*) and surface area (*Lc.S*) of lacuna at 30 nm were respectively 347.8 µm^3^ and 369.7 µm^2^. The average length, width and depth of lacuna (*Lc.L*1, *Lc.L*2 and *Lc.L*3) at 30 nm were 17.2 µm, 9.4 µm, 4.8 µm. All parameters are consistent with previous works^8,31,47,49^. For instance the lacunar volumes *Lc.V* are found in a range varying from 50 to 730 µm^3^ ^15,30^. The lacunar surface was previously reported to be 336.2 ± 94.5 µm^2^ in the work of Dong^31^ and 430.4 ± 68.5 µm^2^ in that of Varga^34^. There are however fluctuations that can be attributed to species or site location.

The present work provides data on the micro porosity related to the LCN in human bone including both the lacunae and the canaliculi network imaged at a very high spatial resolution. The lacunar porosity (*Lc.TV/BV*) was found to be 1.10% and the canalicular porosity (*Ca.TV/BV*) was 0.35%, providing an average LCN porosity of 1.45%. The LCN porosity was underestimated in average by 11% at 120 nm mainly due to the loss of the thinnest canaliculi (see Fig. 4(a,b)). The lacunar porosity has been assessed in human cortical bone in previous works providing a similar range in human tissue^30,31^. There are only few reports on the canalicular porosity, especially measured in 3D in human bones. For instance, Ashique^16^ reported a canaliculi area percentage between 7.9% and 14.3% in human femora but the use of confocal microscopy is likely to overestimate the values. Our values of *Ca.TV/BV* are comparable to those measured from SR imaging reported in previous works 0.57%^34^, 2%^33^. However, Varga^34^ calculated the porosity of canaliculi around specific lacunae instead of covering the whole volume; Hesse^33^ measured porosity in human jaw bone under different health conditions. We also measured the relative ratio of canalicular to lacunar volume, which was found to be 37.7% in average.

We also introduced new parameters to quantify the local porosity of the lacunae and canaliculi within their corresponding Voronoi cells. The average volume of each Voronoi cell (*Cell.V*) was found to be 26000 µm^3^. The average volume of canaliculi per cell (*Ca.V*) at 30 nm voxel size was 115.3 µm^3^. The local porosity of the lacunae and canaliculi (*Lc.V/Cell.V* and *Ca.V*/*Cell.V*) at 30 nm were 1.61% and 0.48%, respectively. These parameters provide data about the density of canaliculi in the neighborhood of each lacuna, giving information about fluid exchanges in this neighborhood.

Another new information from this work is the evaluation of the number of canaliculi per lacuna which was computed at seven distances from the surface of lacuna ranging from 1.2 µm to 12.0 µm. The results show that the average number of canaliculi per lacuna (*Ca.N*) measured at 30 nm increases progressively from 79.7 to 114.2 as shown in Fig. 6(a). This increase is less apparent at 120 nm since the narrowest canaliculi may not be resolved. Varga *et al*. has reported a number of canaliculi per lacuna in the range 53‒126, but using only a fixed distance^34^. Dong *et al*. has also found an increase of the average number of canaliculi per lacuna from 41.5 to 139.1 in the same range of distances^45^. Our results are consistent with this study but our growth rate of *Ca.N* seems to be lower. However, in this previous work, the analysis was performed on a single sample imaged at 300 nm while our study includes 7 samples imaged at 30 nm. The first report of the number of primary canaliculi was an estimation derived from a model and using values of the literature^50^, thus not from direct observations. More recently measurements were reported in rats using confocal microscopy^51^ and ptychography^28^. The ratio between the number of canaliculi per lacuna and the surface area of lacuna (*Ca.N/Lc.S*) describing a local density of canaliculi increases progressively from 0.21 µm^−2^ at 1.2 µm to 0.32 µm^−2^ at 12.0 µm (30 nm voxel size), while on the PCM volumes it fluctuates around 0.16 µm^−2^ (see Fig. 6(b)). These results are consistent with previous reports of the density of canaliculi 0.18 ± 0.03 µm^−2^ or 0.21 ± 0.05 µm^−2^ for mice samples^35,52^. The estimation of the number of canaliculi is an important parameter for the modeling of fluid flow transport and permeability measurements^49,50^.

The measurements at 30 nm were compared to those made on site-matched registered volumes acquired at 120 nm. Concerning the morphological properties of lacunae, the average volume (*Lc.V*) and surface (*Lc.S*) of lacunae at 30 nm, of respectively 347.8 µm^3^ and 369.7 µm^2^, were bigger than those measured at 120 nm (315.6 µm^3^ and 326.0 µm^2^). The average length, width and depth of lacunae (*Lc.L*1, *Lc.L*2, and *Lc.L*3) at 30 nm were slightly larger with differences smaller than 0.5 µm than those evaluated at 120 nm. Nevertheless, we found very high Spearman correlation coefficients (*R*^2^ > 0.9) for all lacunae parameters. Compared to the average size of lacunae, a voxel size of 120 nm is sufficient to measure lacunae properties. The significant differences found on the lacunae volume, surface and sizes are likely to be related to the inclusion of canalicular junctions in the segmented lacunae at 30 nm. This introduces small protuberances on the lacunae surface, which slightly modifies the measurements and could be avoided by smoothing the lacunae surface. Besides, the selected thresholds had impact on the segmented results although we have used hysteresis thresholding to refine the segmentation. The shape and anisotropy of lacunae were the same at 30 nm and 120 nm. The values of *Lc.SMI*, quantifying the global shape of the sample did not change with the resolution and are consistent with previous reports: 3.1^31^. The average ratios between the length, the width and the depth are both close to 4:2:1 for the two resolutions.

The visual observation of the LCN as illustrated on the 3D rendering of Fig. 4 does not permit to appreciate clearly the differences in the network. However, most canaliculi parameters were affected by the decrease of spatial resolution. The quantitative analysis of canaliculi demonstrates clearly that there is a loss in the canaliculi volume and numbers of canaliculi. The ratio *Ca.V/Lc.V* at 30 nm was also higher (37.7%) than the one quantified at 120 nm (34.5%). At all the seven distances, the average number and density of canaliculi (*Ca.N* and *Ca.N/Lc.S*) were significantly different at 30 and 120 nm. This shows that we achieved more accurate segmentation and quantification of canaliculi using the volumes at 30 nm. Thus, the high spatial resolution images yield more accurate investigation results of canaliculi thanks to better resolving power matching their tiny physical diameter (~200‒500 nm)^34,49^.

Although we can better observe and quantify canaliculi at 30 nm, the measurements are limited to a small region of interest (currently 61.4 µm in the three directions). The complementary observation of the LCN in the 120 nm images offering a larger field of view with a volume 64 times larger permits to have more insight on the localization of the VOI analyzed at 30 nm. Most VOIs were located in osteonal tissue but one sample was at the frontier with interstitial tissue, clearly showing a different organization with an obvious decrease of lacunae and canaliculi. Thus, although the images at 120 nm definitely miss canaliculi, they provide a more global observation of the sample. This scale can thus be particularly interesting to analyze organized versus disorganized LCN in human disease or animal models. In perspective, the implementation of techniques such as correlative microscopy could help with the selection of the zoom-in VOI at 30 nm within the larger field of view, and the increase of the detector size could permit to increase the field of view at 30 nm.

This study has some limitations. First, the quantitative results on the LCN were obtained from seven samples, which remains a limited number. Nevertheless, we could observe a large variability in the LCN within this population and there are few comparable data on human tissue in the literature. In perspective, we expect to quantify a larger number of samples from normal and diseased specimens. Second, one limitation related to the imaging technique is that it does not allow seeing the actual cells but only the porous network embedding the cells^22^. However, our images have an isotropic voxel size, which prevents some bias in measurements like it is recognized to happen in confocal microscopy^51^ and the depth of the FOV is not limited by light penetration. Third, although a complete image processing workflow has been used, more research could allow improving the segmentation accuracy of canaliculi, which remains challenging. Therefore, a perspective could be to develop further segmentation methods dedicated to canaliculi by trying to preserve the connectivity of canaliculi without introducing false detections. Finally, one should design additional descriptors to evaluate the properties of the whole network instead of lacunae and canaliculi separately, to better understand the spatial distribution and the topological structure of the LCN.

In conclusion, this work presents an assessment of the lacuno-canalicular in human bone with unprecedented 3D isotropic spatial resolution. The obtained images at 30 nm voxel size provide the most precise measurements to assess the LCN network and the morphology of canaliculi. While the canaliculi parameters are more accurate at 30 nm, a voxel size of 120 nm permits to investigate the 3D properties of the whole network on a larger field of view and may give us apparent parameters that could be meaningful when comparing different populations of samples. Thus, the choice of the 30 nm versus 120 nm voxel size depends on the aim of the study. Overall the technique employed here providing an isotropic spatial resolution may compare favorably to techniques based on visible light imaging. Our results on lacunae are consistent with previous studies and bring direct measurements of canalicular volume, porosity and count of canaliculi stemming from each lacuna, which have not been previously reported, especially on human bone. It is expected that these quantitative parameters will constitute reference values in the field and will contribute to enhance our knowledge on the bone cell network. The presented methodology also opens new perspectives to assess the LCN in bone disease.

## Supplementary information

Legends of supplementary Figures.

Supplementary Video S1:Animation showing the 400 middle slices in volume A1 (at 30nm).

Supplementary Video S2:Animation showing the corresponding 100 middle slices in volume of P1 (at 120nm).

Supplementary Video S3:Animation showing a rotating 3D rendering of the entire segmented volume A1 (at 30nm).

Supplementary Video S4:Animation showing a rotating 3D rendering of the corresponding segmented volume P1 (at 120nm).

Supplementary Table S1:Quantitative parameters computed for all the reconstructed volumes at 30 nm.

Supplementary Table S2:Quantitative parameters computed on the cropped reconstructed volumes at 120 nm.

## Data Availability

The datasets that support the plots within this paper and other findings of this study are available from the corresponding author upon reasonable request.
