# Supplementary material for: Assessment of the human bone lacuno-canalicular network at the nanoscale and impact of spatial resolution
Source: Sci Rep. 2020 Mar 12;10:4567. doi: 10.1038/s41598-020-61269-8 (PMC7067834; doi:10.1038/s41598-020-61269-8)
Supplement: Supplementary file 1 — Legends of supplementary Figures. [file 41598_2020_61269_MOESM1_ESM.docx]

**Assessment of the human bone lacuno-canalicular network at the nanoscale and impact of spatial resolution**

Boliang Yu^1^, Alexandra Pacureanu^2^, Cécile Olivier^1,2^, Peter Cloetens^2^, Françoise Peyrin^1,2,*^

^1^Univ Lyon, INSA Lyon, Université Claude Bernard Lyon 1, UJM-Saint Etienne, CNRS UMR 5220, Inserm U1206, CREATIS, 69621, Lyon, France

^2^ESRF, the European Synchrotron, 38043 Grenoble, France

* Francoise Peyrin: francoise.peyrin@creatis.insa-lyon.fr

Supplementary Figure 1: Plots of the evolution at different distances of the number of canaliculi $Ca.N$ (left) and the density of canaliculi per lacunae surface ${Ca.N}/{Lc.S}$ (right) for all the samples at 30 nm.


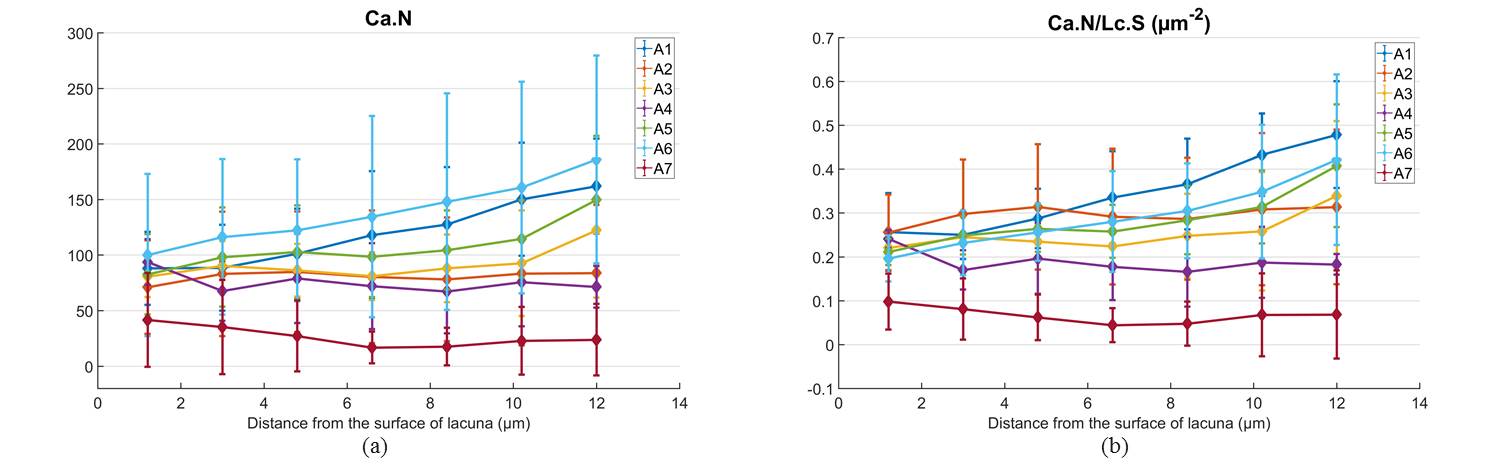


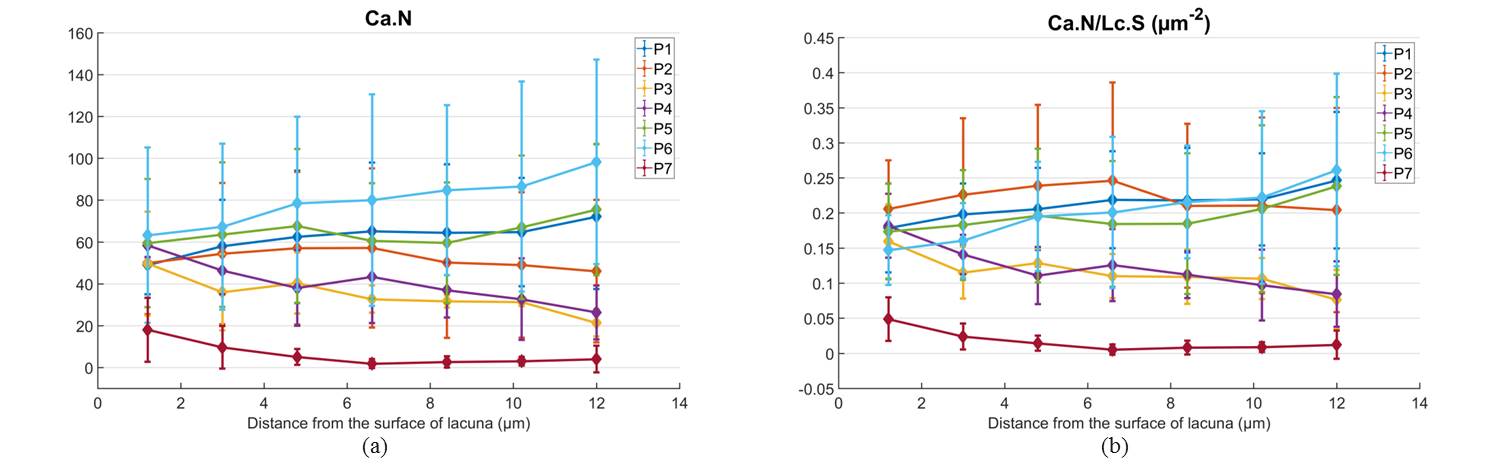


Supplementary Figure 2: Plots of the evolution at different distances of the number of canaliculi $Ca.N$ (left) and the density of canaliculi per lacunae surface ${Ca.N}/{Lc.S}$ (right) for all the samples cropped at 120 nm.
